# Supplementary material for: Short-Term Maize Rotation Suppresses Verticillium Wilt and Restructures Soil Microbiomes in Xinjiang Cotton Fields
Source: Microorganisms. 2025 Aug 22;13(9):1968. doi: 10.3390/microorganisms13091968 (PMC12472141; doi:10.3390/microorganisms13091968)
Supplement: Supplementary file 1 [file microorganisms-13-01968-s001.zip › microorganisms-3764589-supplementary.pdf]

Supplementary Materials

Short-term maize rotation suppresses Verticillium wilt and restructures soil microbiomes in Xinjiang cotton fields

Faisal Hayat Khan[1*], Zhanjiang Tie[1*], Xueqin Zhang[1], Yanjun Ma[1], Yu Yu[2], Sifeng Zhao[1], Xuekun Zhang[1**], Hui Xi[3**]

[1] Key Laboratory of Oasis Agricultural Pest Management and Plant Protection Resources Utilization,
College of Agriculture, , Shihezi University, Shihezi 832003, China Xinjiang Uygur Autonomous Region; fhayat666@gmail.com (F.H.K.); t2424395514@163.com (Z.T.); zhang123451256@163.com (X.Z.); 15630077211@163.com (Y.M.); zhsf_agr@shzu.edu.cn (S.Z.)

[2] Xinjiang Academy of Agricultural Reclamation Sciences, Shihezi 832003, China; xjyuyu021@sohu.com

[3] Workstation of Academician Zhang Xianlong, College of Agriculture, Shihezi University, Shihezi 832003, China

* Correspondence: zhangxk2459@163.com (X.Z.); xihui@shzu.edu.cn (H.X.)

† These authors contributed equally to this work.

Supplementary Table

Table S1 The information of the cotton field sites from northern and southern Xinjiang

Northern fields			Southern fields		
**Field type**	**Location**	**GPS**	**Field type**	**Location**	**GPS**
NCC1	Agricultural Division 125th Regiment 4th Company	Longitude: 44.761558N Latitude: 84.520743E	SCC1	12th Company, 5th Regiment, 1st Division, Aral City	Longitude: 80.758874 Latitude: 41.318358
NCR1	Agricultural Division 125th Regiment 4th Company	Longitude: 44.761558N Latitude: 84.520743E	SCR1	12th Company, 5th Regiment, 1st Division, Aral	Longitude: 80.758514 Latitude: 41.318323
NCC2	Agricultural Division 125th Regiment 11th Company	Longitude: 44.801133N Latitude: 84.54027E	SCC2	13th Company, 11th Regiment, 1st Division, Aral	Longitude: 81.605837 Latitude: 40.550669
NCR2	Agricultural Division 125th Regiment 11th Company	Longitude: 44.801133N Latitude: 84.54027E	SCR2	13th Company, 11th Regiment, 1st Division, Aral City	Longitude: 81.605599 Latitude: 40.550859
NCC3	13th Company, 130 Regiment, Agricultural Seventh Division	Longitude: 44.654653N Latitude: 84.947819E	SCC3	13th Company, 11th Regiment, 1st Division, Aral City	Longitude: 81.612899 Latitude: 40.551702
NCR3	13th Company, 130 Regiment, Agricultural Seventh Division	Longitude: 44.654653N Latitude: 84.947819E	SCR3	13th Company, 11th Regiment, 1st Division, Aral City	Longitude: 81.612807 Latitude: 40.551639
NCC4	Shihezi Academy of Agricultural Sciences	Longitude: 44.336041 Latitude: 86.055469	SCC4	Tiemenguan City 29th Regiment 4th Company	Longitude: 85.958779 Latitude: 41.799565
NCR4	Shihezi Academy of Agricultural Sciences	Longitude: 44.336041 Latitude: 86.055469	SCR4	Provincial Highway 307, Tiemenguan City	Longitude: 85.958073 Latitude: 41.799342
NCC5	Shi Zongchang Sixth Division 11 Company	Longitude: 44.589361N Latitude: 85.984617E	SCC5	Tiemenguan City 29th Regiment 4th Company	Longitude: 85.930761 Latitude: 41.797409
NCR5	Shi Zongchang Sixth Division 11 Company	Longitude: 44.589361N Latitude: 85.984617E	SCR5	Tiemenguan City 29th Regiment 4th Company	Longitude: 85.958786 Latitude: 41.799572
NCR6	133rd Regiment 28th Company	Longitude: 44.802345N	SCC6	Peacock Road, Yuli County, Bayingolin	Longitude: 86.268254 Latitude: 41.319760

		Latitude: 85.058570		Mongolian Autonomous Prefecture	
NCC6	133rd Regiment 28th Company	Longitude: 44.802345 Latitude: 85.058570 E	SCR6	Peacock Road, Yuli County, Bayingolin Mongolian Autonomous Prefecture	Longitude: 86.262889 Latitude: 41.319734
NCR7	134th Regiment 16th Company	Longitude: 44.736616N Latitude: 85.340093E			
NCC7	134th Regiment 16th Company	Longitude: 44.736616 Latitude: 85.340093			

Note: The fields were selected for sample collection, north continuous cropping (NCC), north crop rotation (NCR), and south continuous cropping (SCC) and south crop rotation (SCR) Xinjiang.

Supplementary Figures

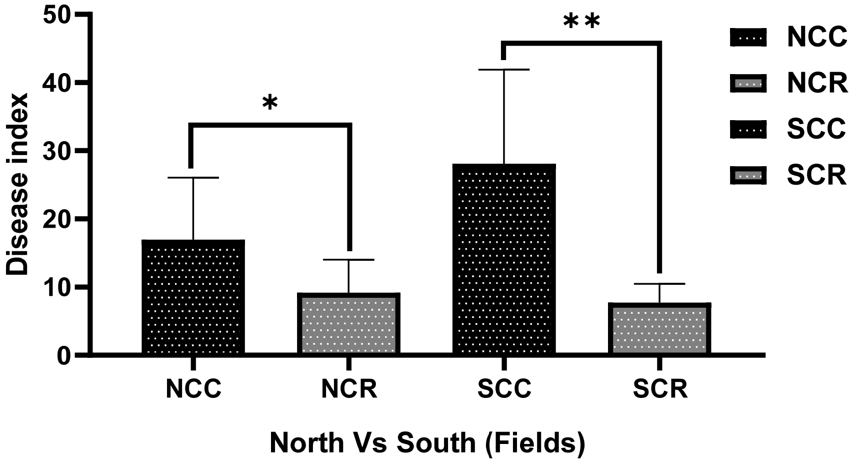

Figure S1. Average disease index of Verticillium wilt under short-term cotton-maize rotation, NCR (North Crop Rotation), NCC (North Continuous Cropping), SCR (South Crop Rotation), and SCC (South Continuous Cropping). Continuous cropping systems (NCC and SCC) exhibit higher average disease indices than their respective rotational counterparts (NCR and SCR), highlighting the potential of crop rotation to mitigate Verticillium wilt severity.

(a)

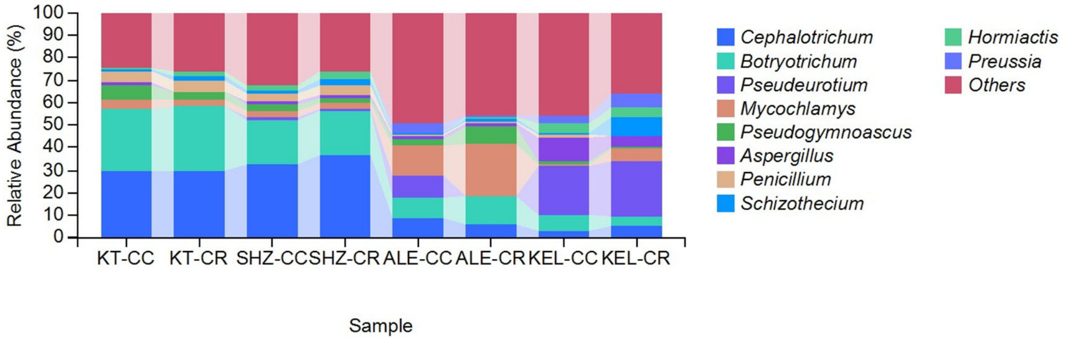

(b)

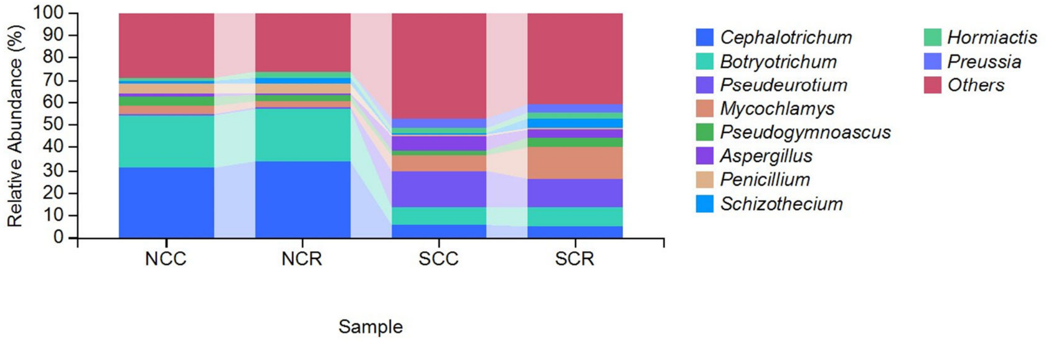

Figure S2. Relative abundance of fungal community in soil samples after cotton-maize rotation (at genus level). (A) Fungal community composition at the genus level in different field sites, comparing continuous cropping (CC) and crop rotation (CR). (B) Aggregated fungal community composition in soils from northern and southern fields under continuous cropping (CC) and crop rotation (CR) regimes.

(a)

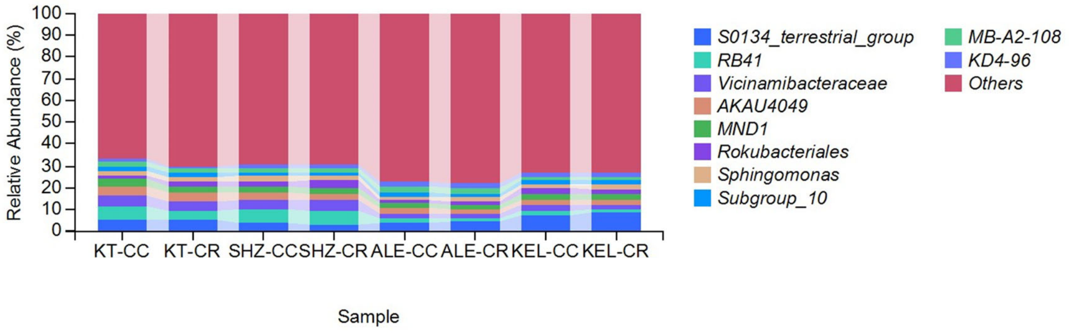

(b)

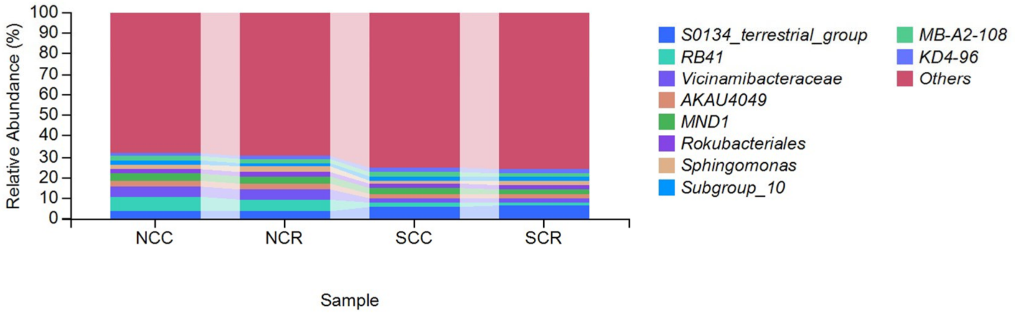

Figure S3. Relative abundance of bacterial community in soil samples after cotton-maize rotation (at genus level). (A) Bacterial community composition at the genus level from different filed sites, comparing continuous cropping (CC) and crop rotation (CR) treatments. (B) Aggregated bacterial community composition in soils from north and south fields under continuous cropping (CC) and crop rotation (CR).

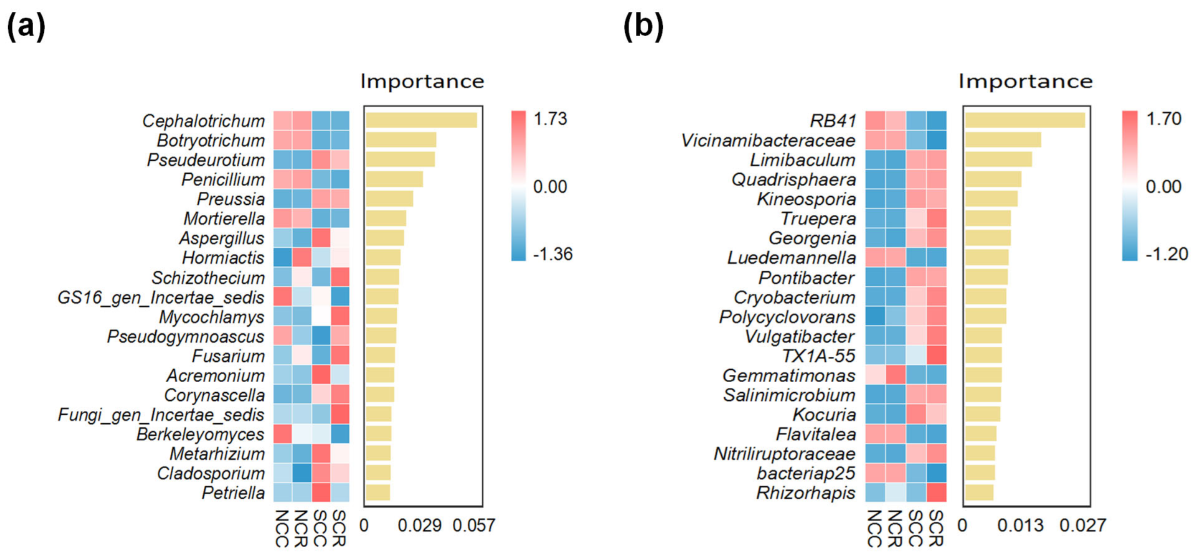

Figure S4. Key taxa associated with different soil treatments based on importance scores. (A) Top fungal genera contributing to community differentiation among continuous cropping and rotation treatments in northern and southern soils (NCC, NCR, SCC, SCR). (B) Top bacterial genera with the highest importance in discriminating between the same treatment groups. Heatmaps indicate standardized abundance (scaled values), while bar plots represent the importance score of each taxon in the model.

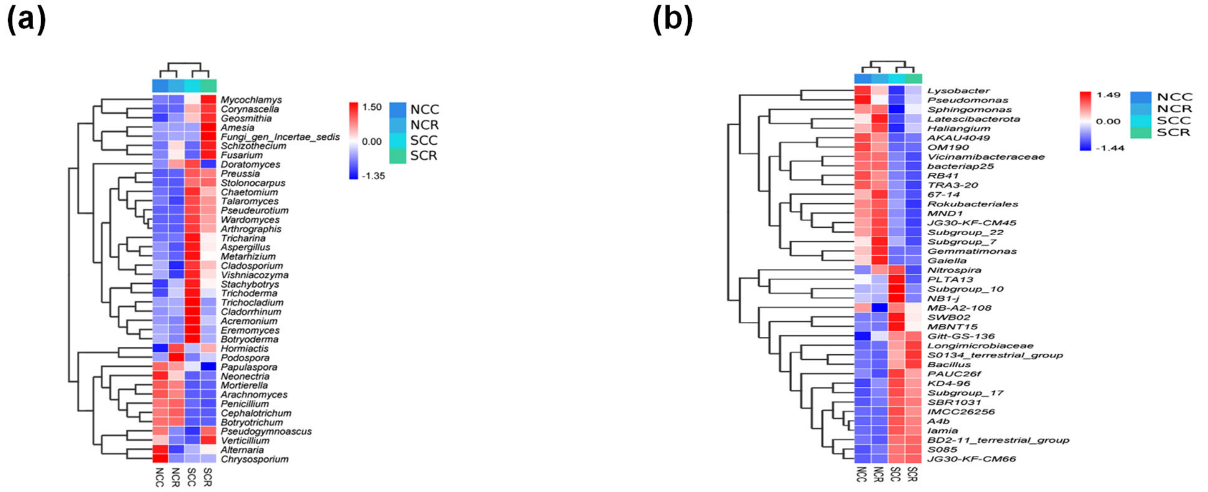

Figure S5. Hierarchical clustering heatmaps of soil microbiome communities based on relative abundance upon cotton-maize rotation in northern and southern fields. (A) Fungal genera showing differential abundance. (B) Bacterial genera showing distinct clustering patterns among treatment groups (NCC), (NCR), (SCC), and (SCR). Color scale represents standardized (z-score) abundance levels, with red indicating higher and blue indicating lower relative abundance.
